# The Causal Effect of Community Hospitals on General Hospital Admissions. Evaluation of a Natural Experiment Using Register Data

**DOI:** 10.5334/ijic.6515

**Published:** 2023-05-03

**Authors:** Terje P. Hagen, Trond Tjerbo

**Affiliations:** 1 Department of Health Management and Health Economics, Institute of Health and Society, University of Oslo, PO Box 1089 Blindern, NO-0317 Oslo, Norway

**Keywords:** cottage hospital, local emergency beds, primary care, health system, hospital services, integrated care, community hospital

## Abstract

**Background:**

To reduce overall healthcare costs, several countries have attempted to shift services from specialist to primary care. This was also the main strategy of the Coordination Reform introduced in Norway in 2012. An important part of the reform was the introduction of Municipal Acute Wards (MAWs), a type of community hospital aimed at reducing admissions to general hospitals. The main objective of this paper is to investigate whether the implementation of MAWs had a causal effect on hospital admissions.

**Methods:**

Monthly admission rates in total and by age groups for patients admitted with acute or elective conditions at internal medicine or surgical departments were analyzed using panel data regression techniques. We identified causal effects by exploiting the sequential roll out of the MAWs within fixed effect analyses. Our data covered all municipalities from start of 2010 until the end of 2017.

**Results:**

The sequential implementation of the MAWs started during the summer of 2012. By the beginning of 2016 close to all municipalities had an operative MAW. The introduction of MAWs significantly reduced acute hospital admissions. The effect was strongest for patients ≥80 years admitted acutely to internal medicine departments. The effects were even stronger if the MAW had a physician on site 24/7 or was located close to a local emergency center.

**Conclusion:**

Our findings suggest that this type of intermediate care unit is a viable option to alleviate the burden on hospitals by reducing acute secondary care admission volumes.

## Introduction

There is an increase in avoidable use of hospitals’ emergency departments and hospital beds among older adults in many countries [1,2]. The problem can result both in misallocation of resources in general and to reduced capacity to treat critically ill patients when needed [3]. Consequently, many countries experiment with reorganizing primary care to reduce the pressure on hospitals. Initiatives include reinventions of community hospitals [4], implementation of observation wards [5] and investments in hospital-at-home- systems [6]. The Norwegian government initialized the so-called Municipal Acute Wards (MAWs), as part of the Coordination Reform in 2012 to reduce undesired or unnecessary admissions to general hospitals [7,8]. The MAWs are organized as an integrated part of the municipal health and care services together with general practitioners (GPs), local emergency services, long term care and social care services [9,10]. Patients presumed to be admitted to MAWs were those who had been assessed by a GP, usually in a GP’s office or at a local emergency service and identified as needing close follow-up and treatment by nurses or primary physicians. MAWs were planned mainly as a service for either stable patients with a known acute primary diagnosis that could be evaluated and treated by primary care methods, or patients whose treatment needed to be re-evaluated and adjusted. Typical MAW patients were expected to be older adults with pneumonia, infections (especially urinary tract infections), gastroenteritis, chronic obstructive pulmonary disease (COPD), diabetes, heart failure or dehydration [8].

To our knowledge, and with the exception of our preliminary analysis [9], no large scale studies have explicitly evaluated the effects of introducing municipal acute inpatient wards on specialist care substitution rates. We therefore examine the effects of the implementation of MAWs on the number of acute and elective hospital admissions within different types of hospital departments, with a special focus on older adults, defined as patients being ≥80 years. The analyses are conducted using data from two years before and five years after the introduction of the Coordination Reform. Information on the exact starting dates and the sequential roll-out of the MAWs permit us to isolate the causal effect of MAWs on the number of hospital admissions.

## Background

### The Coordination reform of 2012

The Norwegian health care system is a two-tier organization: municipalities are responsible for providing primary health care, long-term care, and social services to everyone in need, while the central state is responsible for the specialist services [10,11]. The central state took over the responsibility for specialist health care from the counties in 2002 and organized and funded the services through five, later reduced to four, regional health authorities [12]. The decentralized task structure of the primary care services is combined with central regulations of revenues and service standards. The central state also has the responsibility of exercising supervision and control. Thus, the Norwegian municipalities are more limited in their ability to prioritize and adapt services according to local preferences than suggested in the standard literature of fiscal federalism [13].

Although the Norwegian health care system is generally known for high quality services [14,15,16], a comprehensive reform package named the Coordination Reform was implemented from 2012. The reform came as a response to what the central government defined as three main challenges in the Norwegian health services [7]:

– Patients’ needs for coordinated services were not sufficiently met.
– There was too little initiative aimed at limiting and preventing diseases.
– Population aging and the changing burden of diseases among the population.

The reform can be seen as part of a broader movement towards improving coordination and integration, and shifting of tasks and responsibilities from specialist to primary healthcare [17,18]. The comprehensive policy package involved several measures to ensure successful implementation and commitment of the involved parties [7]. Two new pieces of legislation, The Norwegian Public Health Act (“Folkehelseloven”) of 2012 and the Act of Municipal Health and Care Services (“Helse- og omsorgstjenesteloven”) of 2011, were implemented together with three economic incentive measures:

– An earmarked matching grant to the municipalities to stimulate investments in MAWs,
– municipal co-financing of treatment in the hospitals’ internal medicine departments and outpatient clinics, and
– municipal payment for patients ready for discharge and needing care from the municipalities [19].

The municipal co-financing had no significant effects on hospital admissions and was abandoned from 2014 [20].

The earmarked grant for the MAWs was intended to stimulate investments for a period of four years, from 2012 until 2016, when the MAWs became mandated. From 2016 the earmarked matching grant was included in the central state’s general grant to the municipalities. The municipalities could organize the MAWs either as a municipal service covering one municipality or as inter-municipal cooperations covering two or more (often smaller) municipalities [8,21].

### The MAWs – means to achieve care integration

The MAWs are organizations that aim to increase coordination between primary and specialist care by better triage and by treating selected patient groups at the primary care level. In this way the MAWs can be interpreted as an integrated care initiative with the aim to overcome fragmentation of the system and streamline the clinical pathways [22,23,24].

The MAWs resemble community hospitals known from high-income countries like Australia, Canada, the Netherlands, Sweden, UK, and the US [25,26,27,28]. Winpenny et al’s [29] classification of community hospitals along several dimensions is fruitful also to describe the Norwegian MAWs. First, the MAWs must be distinguished from primary care services without inpatients beds like GPs and local emergency departments. Second, although the MAWs are often co-located with nursing homes, their responsibilities differ. Norwegian nursing homes are primarily occupied by patients in long-term care, yet with increasing amounts of short-term stays related to rehabilitation, respite care and palliative care. The MAWs, on the other hand, provide *acute* inpatient care for patients. Third, like most types of community hospitals, the MAWs are staffed with nurses and generalist doctors (GPs). Fourth, while community hospitals in many countries are located in rural areas, the Norwegians MAWs are present also in *urban* areas. Sixth, unlike in some countries, like Italy, where the focus of community hospitals are on post-acute care [30], the main purpose of the Norwegian MAWs is to reduce specialist care admissions. This means that patients in the MAWs are persons that otherwise would have been admitted to a hospital.

Many of the earlier studies of the effects of community hospitals have been small scale [31,32] or studies with methodological shortcomings [33]. However, several of the case studies found that the introduction of community hospitals and other forms of intermediate care units resulted in a reduction of unnecessary hospital admissions among older adults [31] as well as reductions in prolonged hospital stays and reduction in delayed discharges from the hospitals [34]. A US-study of a post-acute intermediate-care geriatric unit [35] indicate that such units also may represent a potential alternative to acute hospitalization for selected older patients.

Also of importance are studies that have evaluated the effects of comparable initiatives as the MAWs in primary care. Some of these have evaluated the substitution effect of expanding GP and primary care emergency services on the use of hospital emergency capacity. Lowe et al. [36] found that patients’ use of emergency departments was reduced if GP practices were open during the evening hours. Krämer and Schreyögg [2] found negative effects of primary care emergency services on both ambulatory emergency visits and emergency inpatient admissions in a large-scale German study. Both these studies are in their approach similar to ours, but as already indicated, we have not found any quantitative studies that explicitly have evaluated the effects of introducing community hospitals/local acute wards on specialist care substitution rates.

## Theory and Hypotheses

We assume that the municipalities have an objective to cover their citizens’ demand for emergency beds either by MAWs or by using general hospitals. Local authorities and service providers make this demand decision based on information on prices and local supply and demand factors [37,38,39], including information on the patient’s risk profile. Since the number and composition of the individual patients at the MAWs were unknown at the time of the data collection, we applied a simplified demand equation to estimate the effect of the MAWs. The basic hypothesis is that demand for hospital services will be reduced when MAWs are implemented as stated above. Based on the stated objectives of introducing the MAWs, we further expect the following effects:

– The reduction in admissions following the introduction of the MAWs will be greater for older adults than for younger patients.
– The reduction will be higher for acute admissions than for elective admissions. Elective admission should be unaffected or could even increase.
– The reduction in admissions will primarily be at the internal medicine departments, while the activity at surgical departments will hardly be affected.

In an initial analysis based on data from the first two years after its implementation [9], we concluded that the introduction of MAWs was associated with a small, yet significant overall negative effect on acute hospital admissions in internal medicine departments. The reduction was significant for the entire population (–1.2%, 95% CI –2.0% to –0.0%) and slightly stronger for those aged ≥80 years (–1.9%, 95% CI –3.0% to –1.0%). The more detailed analysis of the elderly population aged ≥80 years revealed that effects were dependent on the institutional characteristics of the MAWs, in particular the availability of physicians on site at the MAWs or the Maws’ geographical distance to local emergency centers. In a more in-depth investigation, Nystrøm, Lurås et al. [40] reported that primary care physicians were concerned of using MAWs as an alternative to hospitalisation. These concerns were related to fewer diagnostic opportunities, lower medical expertise throughout the day, uncertainty about the selection of patients and challenges with user participation. Consequently, these concerns had an impact on how the GPs utilised MAW services. This forms the basis for our last hypothesis:

– The reduction in hospital admissions will be higher if the MAW has a physician on site 24/7 or is located close to a local emergency department.

## Data and Methods

### Data and study population

Data on hospital admissions were obtained from the Norwegian Patient Register (NPR) and included patients admitted to hospitals between 1st January 2010 (two years before the start of the Coordination reform) and 31 December 2017, excluding psychiatric hospitals. The patients were grouped along four dimensions:

– Contact types: Admissions or day stays
– Urgency: Acute or elective treatment
– Departments: Internal medicine or surgical
– Age groups: <80 years or ≥80 years

Numbers of admissions for different types of services and age groups were allocated to the municipalities based on the patient’s place of residence, summed, and standardized per 1000 inhabitants. The cut-off at 80 years was pragmatically set but reflects that the MAWs were primarily targeting the population of older adults. As the life expectancy in Norway is above 80 years, a cut-off at 80 will make it possible isolate the effects of the MAWs on the main target group.

We acquired annual municipal data from Statistics Norway’s KOSTRA-database covering all Norwegian municipalities from 2010 through 2017. This database includes variables describing municipal supply of services, such as the number of nursing home beds, number of GPs, GP contracted hours per week in nursing homes, and variables describing the demand for services, i.e., total population and the population of specific age groups. These supply and demand side control variables were normalized per 1000 inhabitants.

We obtained monthly descriptive data for each MAW via structured telephone interviews with the managers of the MAWs or their deputies administered in three rounds taking place during summer 2013, summer 2015 and winter 2018. The data included among other variables describing month of the first MAW admission, available equipment, number of beds and availability of GPs on site. All the data from the data collection used in this article, were based on questions with pre-defined responses.

We linked data by municipality number, year and month followed by linear interpolation to approximate the monthly values for municipal demand and supply data that were reported annually. This gave us a dataset with panel structure. We included all municipalities except four which were amalgamated during the study period (N = 425). The unit of analysis is then municipality-month.

### Statistical model

Our demand model was formalized within a generalized framework for policy analyses of panel data [41]. The coefficients of interest are b_3_ in Equation 1 (Eq. 1) and b_4_ in Equation 2 (Eq. 2).

Eq. 1 {{logDH}_{mt}} = {a_1} + {b_1}log{S_{mt}} + {b_2}log{D_{mt}} + {b_3}MA{W_{mt}} + f + y + t + u

where:

– *logDH_mt_* is the natural logarithm of variables describing use of hospital services for each municipality *m* at time *t* (for further specification, see Table 1),
– *logS_mt_* is a vector of municipal supply variables, i.e., nursing homes and GPs,
– *logD_mt_* is a vector of municipal demand factors, i.e., population and the age composition of the population,
– *MAW_mt_* is a dummy variable that takes the value of 1 in the first month a municipality implements a municipal acute unit and onwards, 0 else,
– *f* are municipality-fixed effects to account for the time-invariant unobserved characteristics of municipalities,
– y are year dummies to account for time specific variation,
– *t* is a seasonal adjuster for month, and
– *u* is the error term for municipality *m* at time *t*.

All variables except the dummies, were standardized by the total population in 1000 in each municipality.

After conducting the analyses of main effects, we dig further into the analyses of the patient group of most interest – the acute admissions among older adults (≥80 years) at internal medicine departments. We described variation in acute preparedness by the variable *PREP_mt_* which is a dummy variable that takes the value of 1 if the MAW has a medical doctor on site 24/7 or is located less than 200 meters from a municipal emergency center, 0 else. The interaction term MAW_mt_*PREP_mt_ will give us the additional effect of having a MAW with acute preparedness as compared to the effect of having a MAW without such a feature. This model can be summarized as:

Eq. 2 \begin{array}{c}
{}{logDH}_{mt} = {a_1} + {b_1}log{S_{mt}} + {b_2}log{D_{mt}} + {b_3}MA{W_{mt}}\\
+ {b_4}MA{W_{mt}}*PRE{P_{mt}} + f + y + t + u
\end{array}

### Analyses

The use of fixed effects in data with panel structure allowed us to control for data heterogeneity and focus on the variable of interest. Of most interest, fixed effects for municipalities give us the within estimator of the *MAW* variables [42].

We regarded all variables except *MAW* as exogenous. *MAW* might have elements of endogeneity as municipalities with high admission rates have a slightly stronger incentive to implement MAWs than municipalities with lower admission rates. We handled this endogeneity problem via municipal fixed effects.

We used SAS, version 14.1, for all analyses.

## Results

### Descriptive statistics

The most common way of organising the MAWs were within nursing homes, where approximately 60% of the units were localised. The size of the units varied from 1 to 72 beds in 2017. Due to the structure of Norwegian municipalities with half of the municipalities having less than 5000 inhabitants, the number of MAWS with less than 3 beds made up 30% of the total. Number of total MAW beds increased from 340 by the end of 2012 to 723 by the end of 2017. In the same period the number of MAW patients increased from app. 6600 to app. 40400 and the number of MAW bed days increased from 16 000 to 104 000 [8,43]. 723 beds in total means that there are 0,13 beds per 1000 inhabitant. For further descriptive analyses, see [43,44,45,46,47].

The main target of the MAWs are the acute admissions at medical departments. These admissions are particularly high among the older adults ≥ 80 years with 32.2 admissions per 1000 inhabitants per year in 2010. Standardized by the total population (Table 1a), the average number per municipality and month is 1.76. Among the younger population (Table 1b) and reflecting the greater size of this age group, the comparable number is 6.04. The number of elective admissions at the medical departments are significantly lower than acute admissions in both age groups. At surgical departments, admissions are divided approximately equally between acute and elective admissions in both age groups. The number of day stays are comparatively low except for elective day stays at surgery departments. There are negative time trends in most of the dependent variables.

**Table 1a:** Average monthly admissions per 1000 inhabitants. Population ≥ 80 years. Weighted by municipal population (2010–2017).


VARIABLES	2010	2011	2012	2013	2014	2015	2016	2017

Acute admissions, Medical departments	1.76	1.72	1.71	1.70	1.70	1.68	1.65	1.66

Acute admissions, Surgery departments	0.31	0.30	0.29	0.29	0.28	0.28	0.29	0.28

Acute day stay, Medical departments	0.07	0.07	0.07	0.07	0.01	0.01	0.01	0.01

Acute day stay, Surgical departments	0.02	0.02	0.02	0.02	0.09	0.08	0.08	0.09

Elective admissions, Medical departments	0.23	0.24	0.24	0.25	0.27	0.27	0.26	0.25

Elective admissions, Surgical departments	0.25	0.25	0.25	0.25	0.27	0.27	0.27	0.27

Elective day stay, Medical departments	0.07	0.05	0.05	0.04	0.01	0.01	0.01	0.01

Elective day stay, Surgical departments	0.36	0.32	0.32	0.30	0.33	0.33	0.34	0.35


**Table 1b:** Average monthly admissions per 1000 inhabitants. Population <80 years. Weighted by municipal population (2010–2017).


VARIABLES	2010	2011	2012	2013	2014	2015	2016	2017

Acute admissions, Medical departments	6.02	5.87	5.80	5.78	5.73	5.75	5.66	5.67

Acute admissions, Surgery departments	1.93	1.88	1.89	1.83	1.80	1.84	1.81	1.80

Acute day stay, Medical departments	0.63	0.62	0.65	0.63	0.03	0.02	0.03	0.02

Acute day stay, Surgical departments	0.32	0.31	0.29	0.28	0.86	0.86	0.84	0.84

Elective admissions, Medical departments	2.03	2.12	2.09	2.12	2.05	2.07	2.05	2.02

Elective admissions, Surgical departments	2.27	2.32	2.31	2.33	2.40	2.38	2.41	2.36

Elective day stay, Medical departments	0.91	0.78	0.71	0.61	0.05	0.04	0.04	0.04

Elective day stay, Surgical departments	3.60	3.64	3.66	3.54	4.10	4.03	3.82	3.80


**Figure 1:**
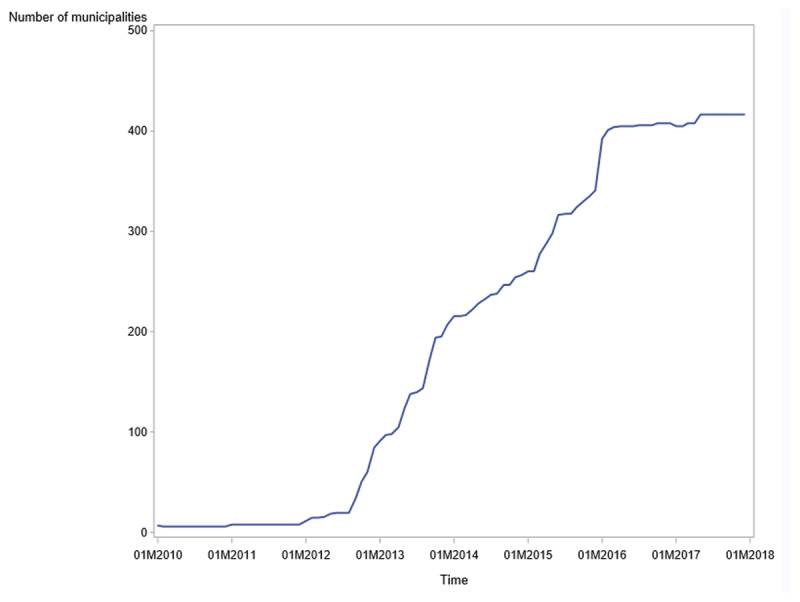
Number of acute wards in Norwegian municipalities, 2010–2017 (N = 425).

By the end of 2014, 221 (51.8%) municipalities had implemented MAWs (Figure 1) and by the beginning of 2016, the time when MAWs became mandatory, all except eight municipalities had implemented MAWs. The growth in MAWs was highest from summer 2012 and the next one and a half year and in the last months of 2015, just before MAWs became mandatory.

Reviewing descriptive statistics for the independent variables (Table 2), we observe a slight reduction in nursing home places over time, while the number of GPs is increasing. The share of population ≥ 80 years was 5.49 percent in 2010 and decreased until 2016. From 2016 to 2017 the relative share of the population ≥ 80 years increased. The population growth in the study period is approximately 1.1 percent per year. Observe that the number of inhabitants in Table 2 with this specification, describes the mean population at municipal level.

**Table 2:** Independent variables. Average per year, 2010–2017. Weighted by municipal population.


INDEPENDENT VARIABLES	2010	2011	2012	2013	2014	2015	2016	2017

Nursing home places (per 1000 inhab.)	11.84	11.84	11.82	11.69	11.56	11.29	11.29	11.31

GPs (per 1000 inhabitants)	0.96	0.96	0.99	1.02	1.02	1.04	1.05	1.05

Population >= 80 years (% of total)	5.49	5.43	5.36	5.30	5.25	5.23	5.22	5.24

Population < 80 years (% of total)	94.51	94.57	94.64	94.70	94.75	94.77	94.78	94.76

Inhabitants	11693	11851	12008	12149	12287	12407	12513	12599

MAW	0.01	0.01	0.05	0.30	0.52	0.69	0.94	0.97

PREP	0.01	0.01	0.04	0.16	0.23	0.33	0.45	0.46


In line with Figure 1, we observe that the share of the population with access to MAW increases from 2012. In 2017, 97 percent of the population had access to MAWs. For approximately half of the population with access to MAW, their MAW either had a medical doctor on site 24/7 or was located less than 200 meters from a municipal emergency centre (the PREP variable).

### Regression results

Our analyses indicate that implementation of MAWs decreased the number of acute admissions at medical departments for the age group ≥80 years by 3 percent (Table 3a). The demand variables yield reasonable effects. A one percent increase in the age group ≥80 years, meaning that the population is getting older, increased the number of acute admissions at hospitals by 0.56 percent. There are also significant and positive effects of number of inhabitants in general. For the variables describing municipal supply of services, effects were small and not always significant. For the elective admissions at medical departments, there were no significant effects of the MAWs. Also, for the other types of services the effects of the MAWs were either insignificant or very small in size (less than 1 percent).

**Table 3a:** Use of hospital services, patients >=80 years. Elasticities from fixed effects analyses.


VARIABLES	ACUTE				ELECTIVE			
	
	ADMISSIONS		DAY STAYS		ADMISSIONS		DAY STAYS	

	MEDICAL DEPARTMENTS	SURGICAL DEPARTMENTS	MEDICAL DEPART-MENTS	SURGICAL DEPARTMENTS	MEDICAL DEPARTMENTS	SURGICAL DEPARTMENTS	MEDICAL DEPARTMENTS	SURGICAL DEPARMENTS

Intercept	–1.50**	–0.63	1.94**	–2.31**	0.62	0.07	8.73**	–9.48**

(log) Nursing home places	0.03*	0.00	0.03**	–0.00	–0.01	–0.02*	0.13**	–0.10**

(log) GPs	0.03	0.00	0.01	–0.00	0.01	0.03*	0.10**	–0.03*

(log) Population >=80 years	0.56**	0.13**	0.08**	0.01	0.09**	0.13**	0.31**	0.03

(log) Population < 80 years	–	–	–	–	–	–	–	–

(log) Inhabitants	0.14**	0.06	–0.20**	0.23**	0.07	0.01	–0.93**	0.94**

MAW	–0.03**	0.00	–0.00*	0.01**	0.00	0.00	–0.01**	0.00

Fixed effects: Municipalities	Yes	Yes	Yes	Yes	Yes	Yes	Yes	Yes

Fixed effects: Year	Yes	Yes	Yes	Yes	Yes	Yes	Yes	Yes

Fixed effects: Month	yes	yes	yes	yes	yes	yes	yes	yes

Adj. R^2^	0.49	0.11	0.34	0.33	0.22	0.18	0.50	0.30


*/** = Significant at 0,05/0,01-level.

For the age group <80 years (Table 3b), the effects of the MAWs were smaller than for the age group ≥80 years. There were, however, negative effects of the MAWs on both acute admissions and acute day stays at the internal medical departments. There were also a few positive effects of the MAWs, in particular on surgical admissions or day stays. In general, the level of these stays was very low (cf. Table 2). The effects of the demographic variables were less stable when analysing the lower age group.

**Table 3b:** Demand for hospital services, patients <80 years. Elasticities from fixed effects analyses.


VARIABLES	ACUTE				ELECTIVE			
	
	ADMISSIONS		DAY STAYS		ADMISSIONS		DAY STAYS	
			
	MEDICAL DEPARTMENTS	SURGICAL DEPARTMENTS	MEDICAL DEPART-MENTS	SURGICAL DEPARTMENTS	MEDICAL DEPART-MENTS	SURGICAL DEPARTMENTS	MEDICAL DEPARTMENTS	SURGICAL DEPART-MENTS

Intercept	10.02**	–37.19**	19.37**	7.73**	–22.84**	33.17**	5.07*	–15.36**

(log) Nursing home places	0.06**	–0.17**	0.07**	0.07**	–0.02	0.17**	0.03**	–0.00

(log) GPs	0.05**	–0.06**	0.10**	0.01	–0.01	0.06**	0.14**	–0.09**

(log) Population >=80 years	–	–	–	–	–	–	–	–

(log) Population < 80 years	–1.43**	6.38**	–2.37**	–2.81**	4.98**	–4.49**	2.40**	2.20**

(log) Inhabitants	–0.18**	0.92**	0.83**	0.54**	0.08	–1.18**	1.65**	0.65**

MAW	–0.01**	–0.00	–0.01**	0.01**	0.00	0.01*	0.02**	0.01**

Fixed effects: Municipalities	Yes	Yes	Yes	Yes	Yes	Yes	Yes	Yes

Fixed effects: Year	Yes	Yes	Yes	Yes	Yes	Yes	Yes	Yes

Fixed effects: Month	Yes	Yes	Yes	Yes	Yes	Yes	Yes	Yes

Adj. R^2^	0.55	0.29	0.54	0.67	0.55	0.55	0.77	0.72


*/** = Significant at 0,05/0,01-level.

Table 4 presents the result of the analyses where interaction terms between MAW and acute preparedness were included.

**Table 4:** Demand for acute hospital admissions at internal medicine departments. Elasticities from fixed effects analyses.


VARIABLES	ADMISSIONS	

	≥80 YEARS	<80 YEARS

Intercept	–1.62**	9.20**

(log) Nursing home places	0.03*	0.06**

(log) GPs	0.03**	0.05**

(log) Population > = 80 years	0.55	–

(log) Population < 80 years	–	–1.25**

(log) Inhabitants	0.15**	–0.18**

MAW	–0.02**	0.01

MAW * PREP	–0.01*	–0.02**

Fixed effects: Municipalities	Yes	Yes

Fixed effects: Year	Yes	Yes

Fixed effects: Month	yes	yes

Adj. R^2^	0.49	0.55


*/** = Significant at 0,05/0,01-level.

For the oldest age group, the effects of the introduction of MAWs on acute admissions in medical departments was minus 2 percent. There was an additional effect of minus 1 percent if the MAW was organized with a physician on site 24/7 or was located close to a local emergency centre. For the age group <80 years, the reduction in acute admissions could be found only among municipalities that had a MAW with the highest level of acute preparedness.

## Discussion

Our analysis revealed effects of the Norwegian Municipal Acute Wards largely in line with expectations. There was an overall significant reduction in acute admissions for patients ≥80 years in internal medicine departments of 3 percent. For acute day-stays, there was a reduction of 1 percent. The reduction in acute admissions to internal medicine departments for patients <80 years was estimated to 1 percent. For elective admissions at internal medicine departments and for both admissions and day-stays at surgical departments, we found no stable effects, which also were in line with our expectations. Interestingly, there was a significant interaction effect between the variable describing the introductions of the MAWs and the variable describing the acute preparedness. For the highest age group, the effect of the introduction of MAWs on emergency admissions in medical departments was minus 2 percent. There was an additional effect of minus 1 percent if the MAWs were organized with a physician on site 24/7 or were located close to a local emergency centre.

The results in this analysis are larger in magnitude than in a former analyses of the effects of the MAWs based on data from the first two years after the implementation of the Coordination reform [9]. The effects at that time for those aged ≥80 years and admitted to internal medicine departments were estimated to minus 1.9 percent, compared to minus 3 percent in the present study. In relative terms, the effect has increased by a bit more than 50 percent, which probably reflects higher admission rates at the MAWs [48].

Community hospitals are intermediate solutions that occupy a space between specialist and primary care [29,30]. The Norwegian MAWs were explicitly designed to be an alternative to specialist care, and their functioning and effects provide us with some indications as to how community hospitals can be organized and what effects and implementation features are likely to be important. The main finding in this article was that the MAWs worked as intended and reduced hospital admissions. There is one important caveat: the effect was strongest for the older adults. For patients <80 years of age, only the MAWs with the highest acute preparedness had an effect. This is important as it indicates that the level of acute services present in the intermediate unit is crucial. It indicates an important area both for future research on community hospitals and the development of intermediate care alternatives to specialist care.

However, the introduction of the MAWs has not been without problems. A former study, Nystrøm, Lurås et al. [40], reported that primary care physicians were concerned of using MAWs as an alternative to hospitalisation. The concerns were related to fewer diagnostic opportunities, lower medical expertise throughout the day, uncertainty about the selection of patients and challenges with user participation. These findings, which represent an important critique of the MAWs, are qualified by our findings of the effects of acute preparedness. The conclusion that there was a stronger effect on hospital admissions of a MAW organized with a physician on site 24/7 or were located close to a local emergency centre, a model that is accessible for half of the population having access to a MAW, indicate however that the concerns of lower medical expertise should be nuanced.

A second potential problem relates to the triage of patients. As reported by Hernes, Baste et al. [49], 24% of the patients referred to a large MAW in the County of Østfold were further transferred to hospital. Most transfers to hospital occurred within 24 hours from admission to the MAW. No unexpected deaths were reported. This is a relative high share of transfers. Yet, if the patients in need of specialized health care are rapidly identified, this is not necessarily a problem. On the contrary, the observation time at MAW might have helped identify a more severe underlying health problem which otherwise might have gone unnoticed. MAWs might not only be an alternative to hospitals, but also a good alternative for patients in need of observation [32]. In the study by Hernes, Baste et al. [49], 42% of the admissions at the MAW were due to need for observation.

It is also observed that the municipalities over time use the MAWs in a more flexible way than intended. At least in some smaller municipalities, patients that are discharged from hospitals now also use the MAWs. In this way the MAWs also become stepdown facilities and their ways of working come closer to the intermediate wards known from other countries [50].

To our knowledge and with the exception of our former preliminary analysis, this study is the first that explicitly quantifies the effect of local acute beds on general hospital admissions. The major strength of the study was data on the exact starting dates of the MAWs that permitted utilizing the sequential roll-out to isolate the causal effects of MAWs on the number of hospital admissions. Unlike other studies that have focused on outcomes and patient satisfaction, this study has demonstrated the significant impact of intermediate care alternatives on general hospital admission rates.

There are a few caveats to consider when evaluating our results. Prior to being mandated, the introduction of MAWs by the municipalities occurred via self-selection, which raises possible concerns regarding endogeneity affecting generalizability. Fixed effects reduce the problems by allowing us to compare effects over time within the same municipalities. A second and more fundamental weakness is that we lacked patient level data from the MAWs. These individual data are available from 2022 (covering the period from 2017 and onwards) and will be carefully analysed in future studies. The availability of individual data from the MAWs will make it possible to link data from these services to data from home services, nursing homes, and hospitals and by this describe the entire pathways of care.

## Conclusion

The MAWs were established with the expectation that intermediate care would promote more suitable and ultimately more cost effective healthcare solutions to acute specialist care, particularly in the older adults. Our findings indicate reduction in hospital admissions through the implementation of the MAWs. The consistent results of our analyses suggest that intermediate care, such as MAWs is a step toward alleviating the burden on hospital care capacity by reducing admission volume. However, acute preparedness is likely a key variable for understanding the effects of these intermediate care alternatives.
